# CD44v/CD44s expression patterns are associated with the survival of pancreatic carcinoma patients

**DOI:** 10.1186/1746-1596-9-79

**Published:** 2014-04-08

**Authors:** Zhonghu Li, Kai Chen, Peng Jiang, Xi Zhang, Xiaowu Li, Zhihua Li

**Affiliations:** 1 Department of Hepatobiliary Surgery Institute, Southwest Hospital, Third Military Medical University, 30 Gaotanyan Street, Shapingba District, Chongqing 400038, China

**Keywords:** CD44, CD44v, CD44s, Pancreatic carcinoma, Survival

## Abstract

**Background and purpose:**

CD44 variants have been associated with tumor invasion and metastasis, but CD44 expression patterns have not been systematically investigated in pancreatic carcinoma. This study systematically investigated whether CD44 expression patterns are involved in pancreatic carcinoma metastasis and prognosis.

**Methods:**

We applied primers specific for all CD44 variants and CD44s to analyze the expression patterns of CD44 (CD44v2-CD44v10 and CD44s) using quantitative real-time PCR (qRT-PCR). We then further evaluated their roles in pancreatic carcinoma metastasis and prognosis using clinical survival analysis.

**Results:**

Increased CD44v expression and decreased CD44s expression were found in metastatic pancreatic carcinoma in three different cell lines and in human tumor tissue. Clinical analysis showed that CD44v6^+^ and CD44v9^+^ were correlated with lymph node metastasis, liver metastasis and TNM stage. However, CD44s^−^ was associated with liver metastasis, tumor differentiation and TNM stage. Survival analysis showed that patients with CD44v6^+^/CD44s^−^ or CD44v6^+^/CD44s^−^ had lower overall survival (OS) rates, although the individual expression of CD44v6, CD44v9 and CD44s was also related to decreased OS rates. Univariate analysis showed that lymph node metastasis; vessel invasion; hepatic metastases; TNM stage; and individual or co-expression of CD44v6, CD44v9 and CD44s were risk factors affecting survival. Multivariate analysis showed that CD44v6^+^/CD44s^−^ was an independent predictor of survival.

**Conclusions:**

We found that CD44v6^+^, CD44v9^+^ and CD44s^−^ were associated with pancreatic carcinoma metastasis and progression and that CD44v6^+^/CD44s^−^ was an independent risk factor affecting survival in pancreatic carcinoma. Therefore, the different expression patterns of CD44v/CD44s may determine pancreatic carcinoma prognosis.

**Virtual slides:**

The virtual slide(s) for this article can be found here: http://www.diagnosticpathology.diagnomx.eu/vs/1579257224116287.

## Background

Pancreatic carcinoma (PCa) is undoubtedly the most aggressive and the deadliest cancer [1]. Early metastasis to regional lymph nodes or hematogenous spread to distant organs is largely responsible for the lowest 5-year survival rate (less than 5%) [2,3]. Numerous molecules have been described and extensively investigated for their potential roles in the tumorigenesis and progression of PCa; CD44 is the most important of these molecules.

CD44 is quite complicated. This molecule is encoded by 20 exons and undergoes extensive alternative splicing to generate CD44s (CD44 standard) and CD44v (CD44 variants) [4-6]. CD44s consists of exons 1–5 and 16–20 and is called the constant form. The variable exons are typically numbered v1-v10 (v1 is not encoded in humans), corresponding to the genomic exons 6–15, and are alternatively spliced and incorporated into the variable region either singly or in combination. This process has the potential to generate thousands of different CD44 isoforms (Additional file 1: Figure S1) [6-8], and as a result, CD44 has complex and diverse functions.

CD44 was initially identified as a lymphocyte homing receptor and transmembrane glycoprotein [4,9] commonly expressed in embryonic stem cells [10] and in hematopoietic and cancer stem cells [11-13]. Although CD44 plays an important role in many cell processes, including growth, differentiation and motility [14], there are discrepancies in the literature about the roles of CD44v and CD44s in tumor progression. In certain cancers, CD44s and CD44v are considered to be tumor progression promoters [15-27], but in other cancers, CD44s and CD44v may be involved in tumor suppression [28-39]. These discrepancies may be the result of different methods for detecting CD44 (such as immunohistochemistry (IHC) or PCR) and identifying different CD44 variants in different tumor types. The roles of CD44 in PCa are still disputed. Tsukuda, H [40] reported that CD44v6 and CD44v2 were expressed in the pancreatic juice of patients with pancreatic carcinoma, but the authors also reported CD44v expression in normal cases. Rall, C. J. [41] studied CD44 expression in 21 clinical PCa tissues and reported the expression of CD44v6, CD44v8-9, CD44v8-10 and CD44s, although only CD44v6 may be involved in tumor metastasis. Tomaszewska, R [42] reported that CD44v6 and CD44s expression was positive in pancreatic carcinoma, and Gotoda, T. et al. [43] reported that CD44v6 and CD44v2 may be useful markers for poor survival after studying the expression of CD44v6, CD44v2 and CD44s via IHC. However, all studies found no significant association between CD44s and tumor progression.

In fact, the expression patterns of CD44 in pancreatic carcinoma have not been systematically investigated at the mRNA level. To investigate whether CD44 expression patterns are related to pancreatic carcinoma metastasis and prognosis, we designed a primer specific for each CD44 variant (CD44v2-CD44v10 and CD44s), detected the expression patterns of CD44 in 101 clinical pancreatic carcinoma samples, and analyzed the relationship of those patterns with the clinical characteristics of pancreatic carcinoma.

## Methods

### Patients and specimens

A total of 101 patients underwent surgery for pancreatic carcinoma at the Department of Hepatobiliary Surgery Institute, Southwest Hospital, Third Military Medical University, China, from January 2008 to January 2010. All patients underwent curative resection by pancreaticoduodenectomy or pylorus-preserving pancreaticoduodenectomy with lymph node dissection. None of the patients received neoadjuvant or adjuvant radio/chemotherapy. All 101 tumor samples were cryopreserved in liquid nitrogen at the time of excision to facilitate RNA extraction and quantitative real-time PCR (qRT-PCR) analysis. The patient characteristics are shown in Table 1. All patients were followed up by radiography, ultrasonography and computed tomography every 3 months after discharge. The median follow-up period was 12 months (range 4–46 months), and during this period, 10 patients were found to have liver metastases, and 1 patient was found to have lung metastases.

**Table 1:** Associations between the status of CD44 variants and CD44s and the categorical clinicopathological parameters of pancreatic carcinoma (n = 101)

**Parameters**	**CD44v2**		**CD44v3**		**CD44v4**		**CD44v5**		**CD44v6**		**CD44v7**		**CD44v8**		**CD44v9**		**CD44v10**		**CD44s**	
	**+**	**-**	**+**	**-**	**+**	**-**	**+**	**-**	**+**	**-**	**+**	**-**	**+**	**-**	**+**	**-**	**+**	**-**	**+**	**-**
Gender																				
P-value	0.379		0.379		0.956		0.68		0.956		0.178		0.956		0.956		0.322		0.379	
Male/Female	40/10	37/14	40/10	37/14	39/12	38/12	39/11	38/13	39/12	38/12	41/9	36/15	38/12	39/12	39/12	38/12	41/10	36/14	40/10	37/14
Age, years																				
P-value	0.875		0.551		0.551		0.777		0.777		0.551		0.551		0.777		0.47		0.551	
<65/≥65	36/14	36/15	37/13	35/16	37/13	35/16	35/15	37/14	37/14	35/15	37/13	35/16	37/13	35/16	37/14	35/15	38/13	34/16	37/13	35/16
Tumor location																				
P-value	0.353		0.796		0.737		0.737		0.796		0.796		0.796		0.737		0.737		0.353	
Head/Body,tail	42/8	46/5	44/6	44/7	45/6	43/7	43/7	45/6	44/7	44/6	44/6	44/7	44/6	44/7	45/6	43/7	45/6	43/7	42/8	46/5
Tumor size, cm																				
P-value	0.9		0.593		0.489		0.346		0.489		0.9		0.9		0.9		0.593		0.271	
≤2/>2	19/31	20/31	18/32	21/30	18/33	21/29	17/33	22/29	18/33	21/29	19/31	20/31	19/31	20/31	20/31	19/31	21/30	18/32	22/28	17/34
Lymphatic invasion																				
P-value	0.625		0.197		0.055		0.197		**0.02**		0.09		0.09		**0.02**		0.129		0.474	
Neg/Pos	27/23	30/21	25/25	32/19	24/27	33/17	25/25	32/19	23/28	34/16	24/26	33/18	24/26	33/18	23/28	34/16	25/26	32/18	30/20	27/24
Vascular invasion																				
P-value	**0.006**		0.408		0.408		0.702		0.951		0.408		0.951		0.702		0.951		0.203	
Neg/Pos	30/20	43/8	38/12	35/16	35/16	38/12	37/13	36/15	37/14	36/14	38/12	35/16	36/14	37/14	36/15	37/13	37/14	36/14	39/11	34/17
Neural invasion																				
P-value	0.875		0.875		0.777		0.3		0.875		0.777		0.551		0.245		0.875		0.47	
Neg/Pos	36/14	36/15	36/14	36/15	37/14	35/15	38/12	34/17	36/15	36/14	35/15	37/14	37/13	35/16	39/12	33/17	36/15	36/14	34/16	38/13
Duodenal invasion																				
P-value	0.295		0.583		0.653		0.653		0.295		0.295		0.653		0.583		0.961		0.147	
Neg/Pos	38/12	43/8	39/11	42/9	40/11	41/9	41/9	40/11	43/8	38/12	38/12	43/8	41/9	40/11	42/9	39/11	41/10	40/10	43/7	38/13
Hepatic metastases																				
P-value	0.484		**0.042**		**0.049**		0.172		**0.049**		**0.049**		**0.042**		**0.049**		**0.049**		**0.049**	
Neg/Pos	44/6	47/4	42/8	49/2	43/8	48/2	43/7	48/3	43/8	48/2	43/7	48/3	42/8	49/2	43/8	48/2	43/8	48/2	48/2	43/8
Differentiation																				
P-value	0.808		0.1		0.507		0.124		0.202		0.927		0.491		0.696		0.428		**0.028**	
Poor	13	16	18	11	17	12	19	10	15	14	14	15	15	14	13	16	16	13	11	18
Middle	32	31	26	37	29	34	27	36	29	34	31	32	29	34	32	31	29	34	31	32
Well	5	4	6	3	5	4	4	5	7	2	5	4	6	3	6	4	6	3	8	1
Stage (UICC)																				
P-value	0.837		0.109		0.057		0.135		**0.008**		0.286		0.061		**0.027**		0.143		**0.028**	
IA/IB	4/11	6/14	3/13	7/12	2/12	8/13	2/13	8/12	2/13	8/12	3/12	7/13	4/12	6/13	4/11	6/14	4/12	6/13	6/18	4/7
IIA/IIB	9/20	6/20	4/22	11/18	5/24	10/16	5/23	10/17	3/25	12/15	5/23	10/17	3/23	12/17	3/25	12/15	4/23	11/17	4/19	11/21
III/IV	0/6	0/5	0/8	0/3	0/8	0/3	0/7	0/4	0/8	0/3	0/7	0/4	0/8	0/3	0/8	0/3	0/8	0/3	0/3	0/8

The bold values indicate P-values less than 0.05, *Neg (−)*: negative, *Pos(+)*: positive, *UICC* Union for International Cancer Control.

This study was approved by the Ethics Committee of Southwest Hospital, and all patients provided written informed consent.

### Cell culture

The Aspc-1, Cfpac-1 and Panc-1 pancreatic carcinoma cell lines were purchased from the Shanghai Biomedical Institute. The Cfpac-1 and Panc-1 cell lines were maintained in Dulbecco’s Modified Eagle’s Medium (DMEM; HyClone, Thermo, USA), and the Aspc-1 cell line was maintained in Roswell Park Memorial Institute 1640 medium (1640; HyClone, Thermo, USA). All cell cultures were supplemented with 10% fetal bovine serum (FBS; GIBCO/Invitrogen, CA, USA) in the presence of 100 U/ml penicillin and 100 mg/ml streptomycin. All cell lines were cultured at 37°C in a humidified atmosphere containing 5% CO_2_.

### Cell invasion assay

To perform invasion assays, 5 × 10^5^ cells in 300 μL serum-free medium were placed in the upper chamber (Millipore, MA, USA) of an insert coated with 30 μL Matrigel (BD, Franklin Lakes, USA), and 600 μL medium (DMEM or 1640) containing 10% FBS was added to the lower chamber. After a 24-hour incubation, the cells remaining on the upper membrane were removed with cotton wool. The cells that had passed through the membrane were stained with hematoxylin, imaged using a BX41 microscope (Olympus, Tokyo, Japan) and counted in 10 fields per well at 200x magnification using a CKX41 inverted microscope (Olympus, Tokyo, Japan). The experiments were independently repeated three times.

### RNA extraction and quantitative real-time PCR

Total RNA was extracted from tissues or cultured cells using RNAiso Plus reagent (TaKaRa, Dalian, China) according to the manufacturer’s protocol. The RNA was stored at −80°C, and reverse transcription (RT) of the extracted RNA was performed using the PrimeScript RT reagent Kit with gDNA Eraser (TaKaRa, Dalian, China). The cDNA was stored at −20°C. qRT-PCR assays were performed to detect CD44 expression using the PrimeScript RT reagent Kit and SYBR Premix Ex Taq (TaKaRa, Dalian, China) according to the manufacturer’s instructions. The results were normalized to the expression of beta-actin. The primers used are shown in Additional file 2: Table S1. For quantitative measurement of the CD44 variable exons expressed, qRT-PCR was performed using a CFX96 Real-Time system (Bio-Rad, CA, USA) with the following cycling conditions: 95°C for 30 sec, followed by 40 cycles of 95°C for 5 sec and 60°C for 30 sec. The qRT-PCR results were analyzed and expressed relative to those of a normal pancreas sample and were then converted to fold changes. The PCR products were separated on a 2.0% agarose gel, stained with ethidium bromide (EB) and photographed with a video camera (Vilber Lourmat, Marne-la-Vallée, France). The sizes of all of the qRT-PCR products for CD44v and CD44s are referred to in Additional file 2: Table S1. All experiments were performed in triplicate, and the means of three values are presented.

### Calculating the optimized cut-off point for CD44v2-CD44v10 and CD44s

As quantitative analysis of CD44v2-CD44v10 and CD44s expression in pancreatic carcinoma is novel, no established cut-off points are available. For this analysis, we used two methods to select the cut-off point: (1) the log-rank test, performed by testing the prognostic value at each possible cut-off point (e.g., 25%, 50%, 75%) and (2) Cox regression analysis, including all possible cut-off points (e.g., 25%, 50%, 75%). We divided the samples into two groups, according to the median and the 25th and 75th percentiles. We found that the median cut-off point gave a lower P-value than did the other points using both the log-rank test and Cox regression analysis, with a comparable hazard ratio (HR) for overall survival (OS). Only CD44v6, CD44v9 and CD44s were significantly associated with OS (data not shown), and only those variants were used in the cut-off point analysis (Additional file 3: Figure S2). Using the median cut-off point, we defined a group with ‘low CD44’ (negative) and a group with ‘high CD44’ (positive). All Chi-square (χ^2^) analysis, Kaplan-Meier (KM) survival analysis and Cox regression analysis were performed using data based on the median cut-off point.

### Statistical analysis

All analyses were performed using SPSS 19.0 software (IBM, Chicago, USA). The correlation between categorical variables was evaluated using the χ^2^ test. Data were analyzed using Student’s t-test if the involved comparison groups followed normal distributions; otherwise, the nonparametric Mann–Whitney test was applied. Univariate analysis was conducted by the KM method (the log-rank test). Multivariate analysis was performed using the stepwise Cox multivariate proportional hazard regression model (forward, likelihood ratio), and the values of variables not in the equation were picked from step 1. P-values <0.05 were considered to be statistically significant, and all statistical analysis was completed under the guidance of experienced experts from the Statistics Department of third military medical university.

## Results

### Increased expression of CD44v and decreased expression of CD44s were associated with the invasion and metastatic potential of PCa

**Figure 1:**
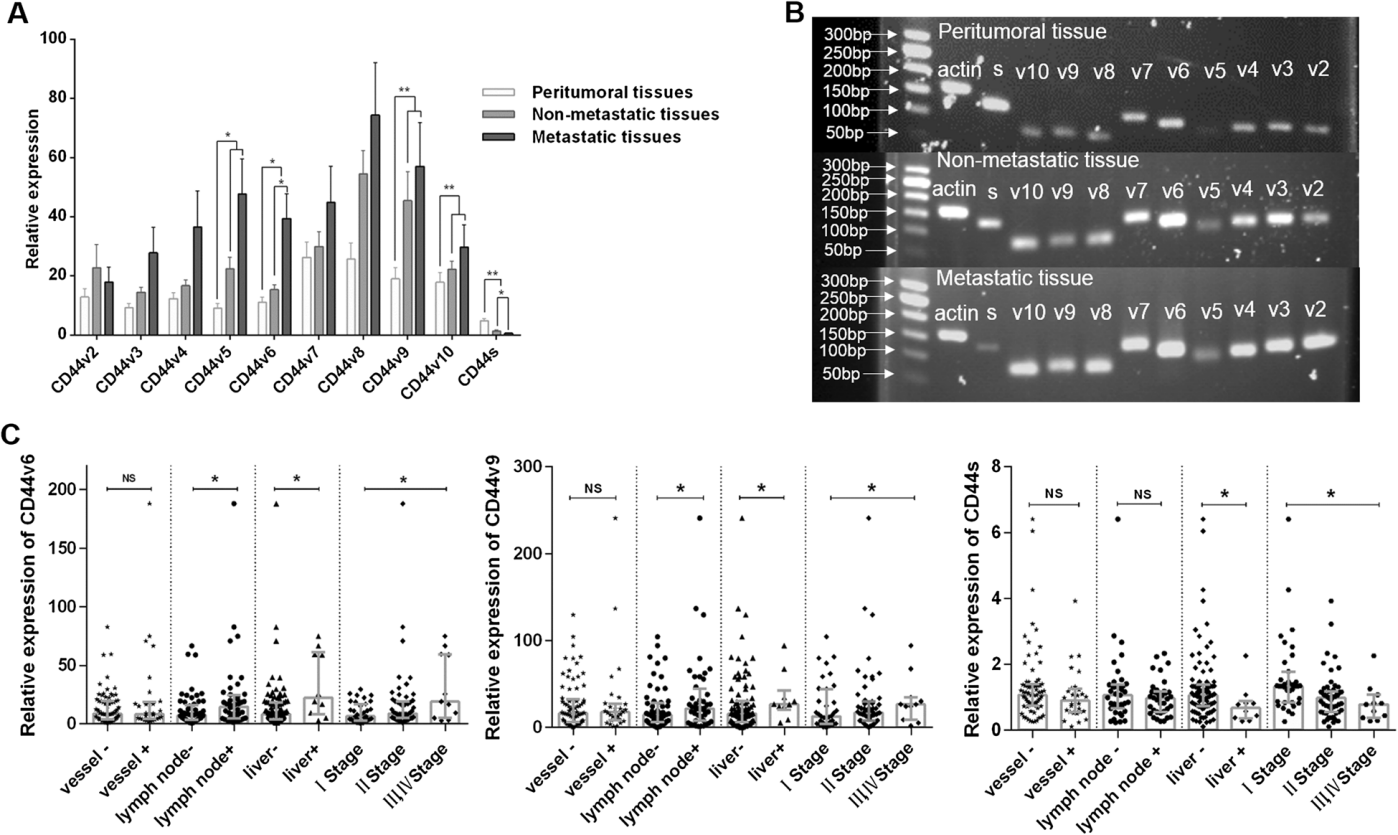
**Expression levels of CD44v and CD44s in clinical pancreatic carcinoma tissues. A** The expression levels of CD44v and CD44s were tested in metastatic tissues (n = 5), non-metastatic tissues (n = 5) and their corresponding peritumoral tissues (n = 10). **B** The PCR products of tissues were confirmed by agarose gel electrophoresis. **C** The expression levels of CD44v6, CD44v9 and CD44s measured by real-time PCR were associated with the clinical characteristics of pancreatic carcinoma (n = 101).

To analyze the expression levels of all CD44 variants, we first designed primers for each variant. Due to the complexity of CD44 isoform expression, we hoped to find a novel method for studying CD44, rather than using previous methods of analyzing CD44 isoforms. The primers for CD44v2-CDv10 were therefore designed to be located in each exon, rather than in exons 1–5 and 16–20, as had been performed previously. The sense primer (3′) for CD44s was located in exon 5, and the antisense primer (5′) spanned exons 5 through 16, producing only CD44s, as described by Konig, H [44] (Additional file 1: Figure S1, Additional file 2: Table S1). All products were verified by agarose electrophoresis (Figure 1B).

**Figure 2:**
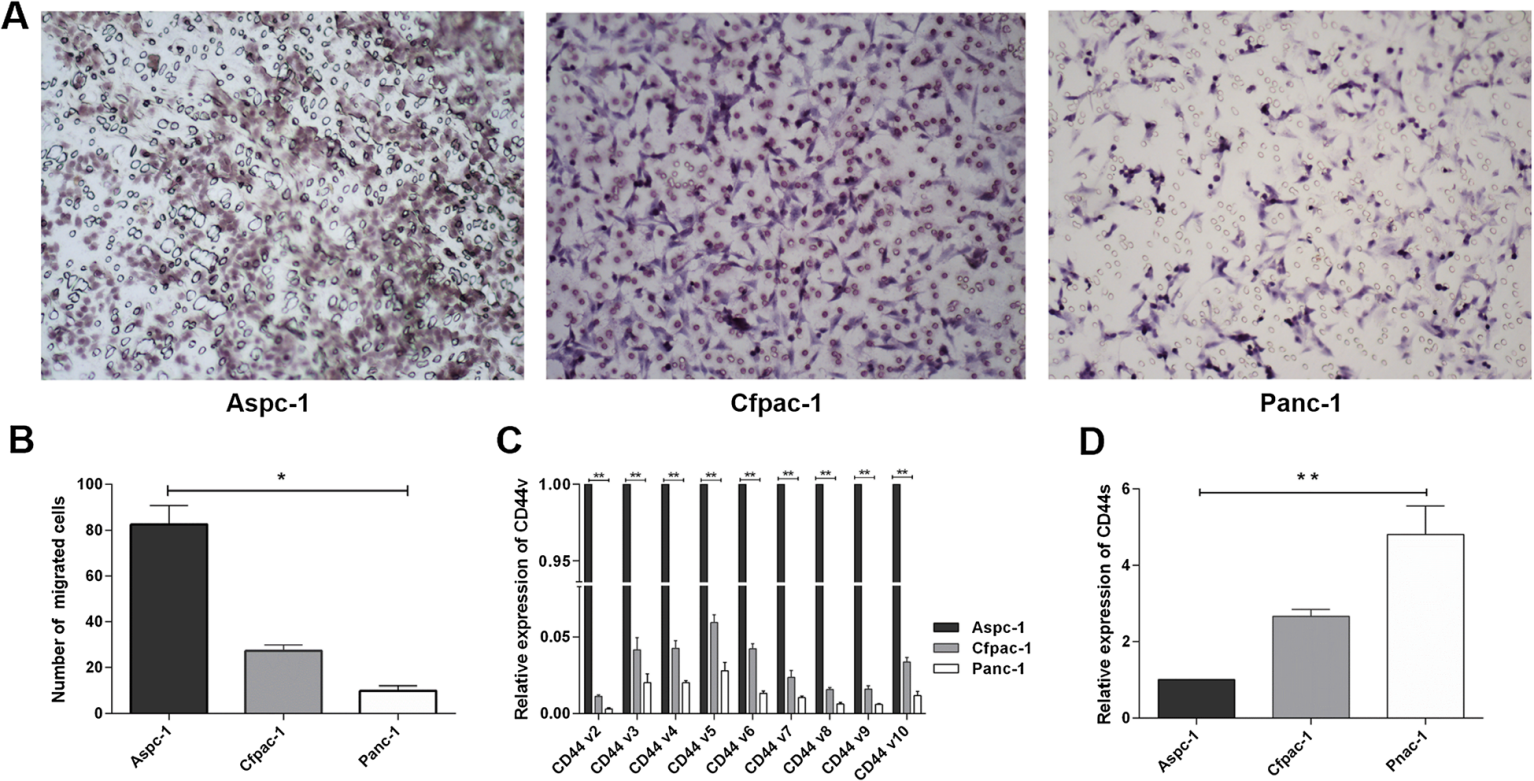
**Invasion capacities and CD44 expression levels of Aspc-1, Cfpac-1 and Panc-1. A-B** Tumor cell invasion activities were measured using a transwell assay. Representative image fields of invasive cells on the membrane are shown. **C-D** Expression levels of CD44v and CD44s in three PCa cell lines.

We then detected the expression levels of all CD44 variants in the three pancreatic carcinoma cell lines. Among the Aspc-1 (As), Cfpac-1 (Cf) and Panc-1 (Pa) pancreatic carcinoma cell lines, the expression levels of CD44v2-CD44v10 (CD44v), from highest to lowest, were in As, Cf and Pa. Interestingly, the expression levels of CD44s, from highest to lowest, were in Pa, Cf and As (Figure 2C, D), which indicates that the pancreatic carcinoma cell lines expressed different levels of CD44v and CD44s.

We next tested the cell invasion capabilities of these three pancreatic carcinoma cell lines using transwell assays. The As cell line showed the highest invasion capacity; Cf, the median invasion capacity; and Pa, the lowest invasion capacity (Figure 2A, B). These results suggest that increased expression of CD44v or decreased expression of CD44s is associated with the invasion potential of the PCa cell lines.

To further explore the relationship between the expression of CD44 and PCa metastasis, we analyzed the expression levels of CD44v and CD44s in five metastatic pancreatic carcinoma samples (which included two hepatic metastases, two lymphatic invasions and one vascular invasion), five non-metastatic samples and ten peritumoral tissue samples. As shown in Figure 1A, when compared with peritumoral tissue, PCa showed both increased CD44v expression and decreased CD44s expression (CD44v5, v6, v9 and v10 and CD44s showed significant differences). Moreover, higher expression of CD44v and lower expression of CD44s were found in metastatic samples compared with non-metastatic samples (CD44v6 and CD44s showed significant differences). These results are consistent with the observations noted when analyzing the three cell lines, which suggests that the expression of CD44v and CD44s is associated with tumor metastasis.

### CD44v6^+^, CD44v9^+^ and CD44s^−^ may be associated with poor prognosis in pancreatic carcinoma

To further investigate whether CD44v2-CD44v10 and CD44s are involved pancreatic carcinoma metastasis, we analyzed the relationship between the clinical characteristics of pancreatic carcinoma patients and the expression of CD44v2-CD44v10 and CD44s. As shown in Table 1, CD44v6^+^ and CD44v9^+^ were significantly correlated with lymph node metastasis (P = 0.02), liver metastasis (P = 0.049) and TNM stage (P = 0.008 and P = 0.027, respectively), and CD44s^−^ was significantly correlated with liver metastasis (P = 0.049), differentiation (P = 0.028) and TNM stage (P = 0.028). However, CD44v2-CD44v5^+^, CD44v7^+^, CD44v8^+^ and CD44v10^+^ were not correlated with any clinicopathological characteristics of pancreatic carcinoma. We further applied a nonparametric Mann–Whitney test to analyze the relationships between the clinicopathological characteristics and the expression of CD44v6, CD44v9 and CD44s. As shown in Figure 1C, CD44v6^+^ and CD44v9^+^ were correlated with lymph node metastasis, liver metastasis and TNM stage, and CD44s^−^ was associated with liver metastasis and TNM stage. These results indicate that CD44v6^+^, CD44v9^+^ and CD44s^−^ may be associated with poor prognosis in pancreatic carcinoma patients.

### CD44v6^+^, CD44v9^+^ and CD44s^−^ are key factors that affect the survival of pancreatic carcinoma patients

**Figure 3:**
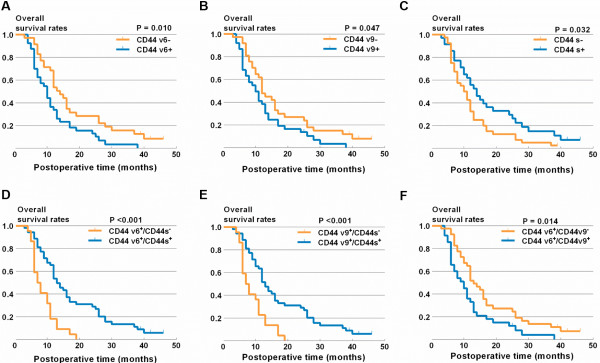
KM survival curves for the OS of patients with pancreatic carcinoma according to CD44v6 (A), CD44v9 (B), CD44s (C), CD44v6/CD44s (D), CD44v9/CD44s (E) and CD44v6/CD44v9 (F).

We further analyzed whether the expression levels of CD44v2-CD44v10 and CD44s were associated with the survival rate of patients with pancreatic carcinoma. KM curve analysis showed that patients with CD44v6^+^ and CD44v9^+^ had significantly low survival rates (P = 0.01 and P = 0.047, respectively). In contrast, patients with CD44s^−^ had significantly low survival rates (P = 0.032) (Figure 3A-C). Patients with CD44v2-CD44v5^+^, CD44v7^+^, CD44v8^+^ and CD44v10^+^ demonstrated no differences in survival rate (data not shown). Interestingly, pancreatic carcinoma patients with tumors that were CD44v6^+^/CD44s^−^ or CD44v9^+^/CD44s^−^ had a significantly lower survival rate than did patients with tumors expressing CD44v6^+^, CD44v9^+^ or CD44s^−^ alone. However, CD44v6^+^/CD44v9^+^ did not further decrease the patients’ survival rate (Figure 3D-F). These results suggest that CD44v6^+^, CD44v9^+^ and CD44s^−^ are key factors affecting the survival of pancreatic carcinoma patients.

### CD44v6^+^/CD44s^−^ expression was an independent risk factor associated with the OS rate of pancreatic carcinoma patients

We used univariate and multivariate analyses to identify independent risk factors associated with the survival rate of pancreatic carcinoma patients. As shown in Tables 2 and 3, univariate analysis revealed that lymph node metastasis (pN); vessel invasion (pV); hepatic metastases; TNM stage; and individual or co-expression of CD44v6^+^, CD44v9^+^ and CD44s^−^ were risk factors that significantly affected the survival of pancreatic carcinoma patients. However, multivariate analysis showed that only D44v6^+^/CD44s^−^ and pN were independent risk factors affecting the OS of pancreatic carcinoma patients (P = 0.003 and P = 0.002, respectively). Therefore, among all CD44 variants, CD44v6^+^/CD44s^−^ was an independent risk factor affecting the OS rate of pancreatic carcinoma patients.

**Table 2:** Univariate analysis of prognostic factors in pancreatic adenocarcinoma (n = 101)

**Variables**	**n**	**Median survival time (months)**	**2-year survival rate (%)**	**P-value**
Age (years)				0.545
< 65	53	12	20.0	
≥65	22	13	20.2	
Gender				0.793
Female	16	10	12.5	
Male	59	12	22.5	
Tumor size (cm)				0.230
≤2	28	12	28.0	
>2	47	11	15.9	
pN				**0.002**
Negative	39	14	28.2	
Positive	36	10	10.8	
Pv				**0.045**
Negative	55	12	26.1	
Positive	20	10	5.0	
Hepatic metastases				**0.000**
Negative	68	12	22.3	
Positive	7	6	0	
Differentiation				0.989
Poor	21	11	25.8	
Moderate	49	12	17.7	
Well	5	10	20.0	
pStage (UICC)				**0.000**
I	24	16	33.3	
II	44	11	15.9	
III,IV	7	6	0	
CD44v6 expression				**0.010**
Negative	35	14	28.5	
Positive	40	10	13.0	
CD44v9 expression				**0.047**
Negative	37	12	27.0	
Positive	38	10	13.7	
CD44s expression				**0.032**
Negative	40	10	12.5	
Positive	35	14	29.6	
CD44v6,v9 expression				**0.014**
Negative	40	13	27.3	
Positive	35	10	12.0	
CD44v6,s expression				**0.000**
Negative	53	13	29.0	
Positive	22	7	0	
CD 44v9,s expression				**0.000**
Negative	53	13	29.0	
Positive	22	7	0	

The bold values indicate P-values less than 0.05. *pN* pathological node stage, *pV* pathological vessel status, *pStage* TNM stage (UICC), *s* CD44s.

**Table 3:** Multivariate analysis of the prognostic factors associated with survival in pancreatic adenocarcinoma (n = 101)

**Independent factors**	**Univariate P**	**Multivariate P**	**Hazard ratio**	**95% Confidence interval**
Pn (Neg/Pos)	0.002	**0.002**	2.313	1.376-3.889
pv (Neg/Pos)	0.045	0.370	1.342	0.705-2.557
Hepatic metastases (Neg/Pos)	0.000	0.051	2.558	0.998-6.555
pStage (I/II/III, IV)	0.000	0.925	1.041	0.450-2.407
CD44v6 expression (Neg/Pos)	0.010	0.281	1.869	0.600-5.824
CD44v9 expression (Neg/Pos)	0.047	0.718	0.748	0.155-3.603
CD44s expression (Neg/Pos)	0.032	1.000	1.000	0.437-2.287
CD44v6,v9 expression (Neg/Pos)	0.014	0.809	0.809	0.146-4.486
CD44v6,s expression (Neg/Pos)	0.000	**0.003**	2.602	1.390-4.870
CD44v9,s expression (Neg/Pos)	0.000	0.810	0.808	0.142-4.587

The bold values indicate P-values less than 0.05 in multivariate analysis, *pN* pathological node stage, *pV* pathological vessel status, *pStage* TNM stage (UICC), *s* CD44s, *Neg* negative, *Pos* positive.

## Discussion

CD44 has been studied for three decades, with hundreds of papers devoted to cancer research, but no consensus opinion on cancer progression has been reached until now. Thousands of CD44 isoforms can be generated due to alternative splicing, and each isoform may function differently. Traditional methods for studying CD44 at the mRNA level make it too difficult to distinguish between splicing isoforms and to analyze clinical tissues. In the present study, we designed primers specific for each exon of CD44 variants and for the exons of CD44s. We could therefore effectively distinguish each CD44 variant in pancreatic carcinoma tissue, rather than trying to identify thousands of complicated CD44 isoforms. A similar method was also applied in Slominski’s research [45]. Although the CD44 variants were distinguished only at mRNA level and protein-level validation of these data is required, but there are no enough specific antibodies to recognize each CD44 variant at present. Therefore, this method is also effective and useful for identifying CD44 splicing isoforms.

Many factors such as lymphatic vascular density, vascular endothelial growth factors (VEGF) and CD44 are involved in PCa progression [46-48]. Our results suggest that increased expression of CD44v and decreased expression of CD44s are associated with pancreatic carcinoma metastasis and progression. First, we found that CD44v expression was increased and that CD44s expression was decreased in pancreatic carcinoma cell lines and in human tissue samples. Second, the individual expression of CD44v and CD44s was associated with invasion and metastasis in pancreatic carcinoma cell lines and in resected PCa tissue. Our results indicate that CD44v and CD44s may play different roles in pancreatic carcinoma metastasis. These results are consistent with the findings of previous studies, which suggested that CD44v and CD44s function differently in pancreatic carcinoma than in other cancers [41,43,49].

CD44v6 and CD44v9 have been identified as markers for tumor progression and metastasis in various cancers [50-53]. However, no consensus has been reached regarding the role of CD44s. The results of the present study indicate that CD44v6^+^, CD44v9^+^ and CD44s^−^ may affect the prognosis of pancreatic carcinoma. CD44v6^+^ and CD44v9^+^ were significantly correlated with lymph node invasion, liver metastasis and TNM stage, and CD44s- was correlated with liver metastasis, tumor differentiation and TNM stage. CD44v2-CD44v5^+^, CD44v7^+^, CD44v8^+^ and CD44v10^+^ were not correlated with any clinicopathological characteristics of pancreatic carcinoma. A nonparametric test showed similar results. Therefore, in pancreatic carcinoma, CD44v6^+^, CD44v9^+^ and CD44s^−^ may be related to poor prognosis.

CD44 is a novel type of molecule that may be involved in tumor growth, invasion and metastasis. A recent study showed that the switch of CD44s to CD44v6 can promote the development of a normal mammary gland into carcinoma [54]. Furthermore, it has been shown that CD44v6 and CD44v9 can increase invasion and metastasis via cooperating with c-Met to activate the MEK and Erk signaling pathways [55] and can increase antiapoptotic ability via inhibiting Fas signaling [56]. In the present study, we found that CD44v6^+^, CD44v9^+^ and CD44s^−^ significantly decreased the survival rate of pancreatic carcinoma patients. Additionally, patients with PCa and co-expression of CD44v6 and CD44s or co-expression of CD44v9 and CD44s had a significantly lower survival rate in comparison with patients with expression of CD44v6^+^, CD44v9^+^ or CD44s^−^ alone. Furthermore, CD44v6^+^/CD44s^−^ was found to be an independent risk factor.

## Conclusions

In this study, we applied a specific method for testing the expression of CD44v2-CD44v10 and CD44s in 101 pancreatic carcinoma tissues, and then further evaluated their roles in pancreatic carcinoma metastasis and prognosis using clinical survival analysis. We demonstrated that among all CD44 variants, CD44v6^+^, CD44v9^+^ and CD44s^−^ were significantly associated with the metastasis and prognosis of pancreatic carcinoma. The different expression patterns of CD44v and CD44s may be useful markers for predicting the prognosis of pancreatic carcinoma.

## Competing interests

The authors declare no conflict of interest.

## Authors’ contributions

ZHL and XWL designed the study, ZHL and KC carried out the experiments and drafted the manuscript, PJ and XZ participated in the statistical analysis. All authors read and approved the final manuscript.

## Supplementary Material

Additional file 1: Figure S1 Schematic of primers specific for CD44v **(A)** and CD44s **(B)** (v2-v10 are abbreviations for CD44v2-CD44v10). Click here for file

Additional file 2: Table S1 Sequences of the CD44v2-CD44v10, CD44s and beta-actin PCR primers. Click here for file

Additional file 3: Figure S2 Identification of cut-off points for CD44v6, CD44v9 and CD44s. Population (n = 101) was divided into two groups according to the median and the 25th and 75th percentiles. P-values and HRs were calculated (Cox regression) for OS for each cut-off point. Click here for file
