# Supplementary figures and images for: CD44v/CD44s expression patterns are associated with the survival of pancreatic carcinoma patients
Source: Diagn Pathol. 2014 Apr 8;9:79. doi: 10.1186/1746-1596-9-79 (PMC4108087; doi:10.1186/1746-1596-9-79)

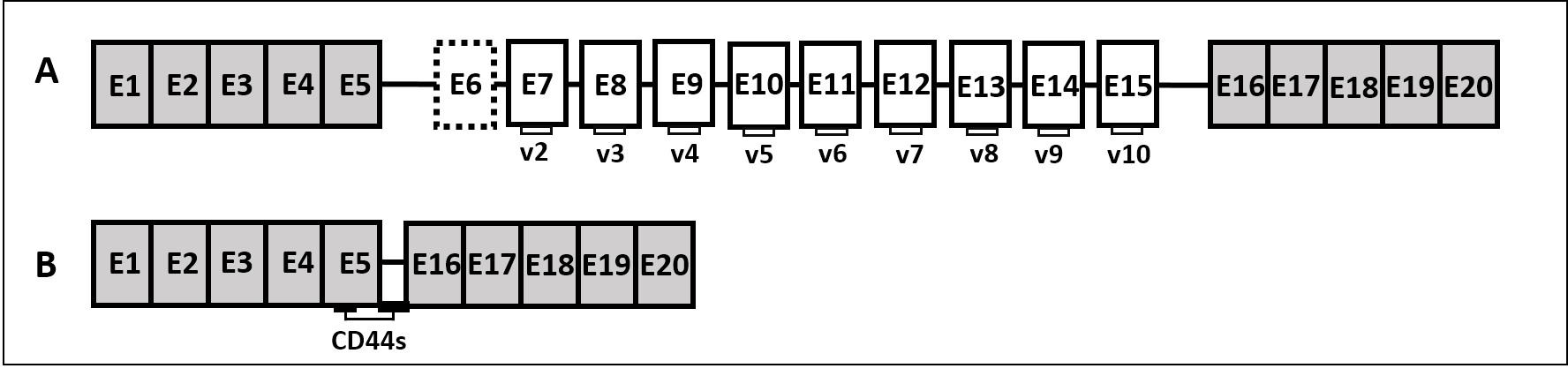

Supplement: Additional file 1: Figure S1 — Schematic of primers specific for CD44v (A) and CD44s (B) (v2-v10 are abbreviations for CD44v2-CD44v10). [file 1746-1596-9-79-S1.tiff]
