# Supplementary material for: CD44v/CD44s expression patterns are associated with the survival of pancreatic carcinoma patients
Source: Diagn Pathol. 2014 Apr 8;9:79. doi: 10.1186/1746-1596-9-79 (PMC4108087; doi:10.1186/1746-1596-9-79)
Supplement: Additional file 2: Table S1 — Sequences of the CD44v2-CD44v10, CD44s and beta-actin PCR primers. [file 1746-1596-9-79-S2.doc]

**Table S1** Sequences of the CD44v2-CD44v10, CD44s and beta-actin PCR primers

|  | Forward Primer | Reverse Primer | Size(bp) | EFF% |
| --- | --- | --- | --- | --- |
| CD44s | GGAGCAGCACTTCAGGAGGTTAC | GGAATGTGTCTTGGTCTCTGGTAGC | 129 | 92.4 |
| CD44v2 | ATCACCGACAGCACAGACAGAAT | AACCATGAAAACCAATCCCAGG | 110 | 96.3 |
| CD44v3 | TACGTCTTCAAATACCATCTCAGCA | AATCTTCATCATCATCAATGCCTG | 110 | 101.3 |
| CD44v4 | AACCACACCACGGGCTTTTG | TCCTTGTGGTTGTCTGAAGTAGCA | 104 | 96.4 |
| CD44v5 | TGCTTATGAAGGAAACTGGAAC | TGTGCTTGTAGAATGTGGGGT | 94 | 96.9 |
| CD44v6 | CCAGGCAACTCCTAGTAGTACAACG | CGAATGGGAGTCTTCTTTGGGT | 112 | 94.9 |
| CD44v7 | GCCTCAGCTCATACCAGCCATC | TCCTTCTTCCTGCTTGATGACCT | 127 | 97.0 |
| CD44v8 | TGGACTCCAGTCATAGTATAACGC | GGTCCTGTCCTGTCCAAATC | 82 | 100.8 |
| CD44v9 | AGCAGAGTAATTCTCAGAGC | TGATGTCAGAGTAGAAGTTGTT | 86 | 92.9 |
| CD44v10 | CCTCTCATTACCCACACACG | CAGTAACTCCAAAGGACCCA | 81 | 100.0 |
| Beta-actin | GTGAAGGTGACAGCAGTCGGTT | GAAGTGGGGTGGCTTTTAGGAT | 157 | 90.7 |

*EFF%*: amplification efficiency
